# Supplementary material for: Direct Photochemical Synthesis of Substituted Benzo[b]fluorenes
Source: Org Lett. 2024 Nov 25;26(48):10364–8. doi: 10.1021/acs.orglett.4c03978 (PMC11629382; doi:10.1021/acs.orglett.4c03978)
Supplement: Supplementary file 2 — ol4c03978_si_002.zip [file ol4c03978_si_002.zip › FID for Publication/6a/20240814_RC-2-C-6-141B_01/plots/RC-2-C-6-141B_gHMBCAD_20240814_01_plot01.pdf]

**RC-2-C-6-141B**

| | | | | | | | |
|---|---|---|---|---|---|---|---|
| Location | 16 | Pulse sequence | gHMBCAD | Temperature | 25 | Suboperator | RuairiCrawford |
| Date collected | 2024-08-14 | Solvent | acetone | Spectrometer | localhost.localdomain-vnmrs500 | Operator | MBaumann |

**SAMPLE**

| | |
|---|---|
| date | Aug 14 2024 |
| solvent | acetone |
| sample | 20240814_RC-2-C-6-141B_01 |

**ACQUISITION**

| | |
|---|---|
| sw | 5341.9 |
| at | 0.150 |
| np | 1602 |
| fb | 4000 |
| ss | 32 |
| d1 | 1.000 |
| nt | 8 |

**2D ACQUISITION**

| | |
|---|---|
| sw1 | 30154.5 |
| ni | 256 |
| phase | arrayed |

**PRESATURATION**

| | |
|---|---|
| satmode | n |
| wet | n |

**TRANSMITTER**

| | |
|---|---|
| tn | H1 |
| sfrq | 499.724 |
| tof | -236.1 |
| tpwr | 58 |
| pw | 6.300 |

**DECOUPLER**

| | |
|---|---|
| dn | C13 |
| dof | 1285.1 |
| dm | nnn |
| decwave | W40_P7374 |
| dmf | 32258 |
| dpwr | 43 |
| pwxlvl | 62 |
| pwx | 14.000 |

**HMBC**

| | |
|---|---|
| j1xh | 146.0 |
| jnxh | 8.0 |

**ADIABATCIC**

| | |
|---|---|
| pwx180ad | P7374_ad300 |
| pwxlvl180 | 57 |
| pwx180 | 465.4 |

**FLAGS**

| | |
|---|---|
| hs | nn |
| sspul | y |
| PFGflg | y |
| hsglvl | 5736 |

**SPECIAL**

| | |
|---|---|
| temp | 25.0 |
| gain | 30 |
| spin | 0 |

**GRADIENTS**

| | |
|---|---|
| gzlvl1 | 478 |
| gt1 | 0.001000 |
| gzlvl3 | 1434 |
| gt3 | 0.001000 |
| gstab | 0.000500 |

**F2 PROCESSING**

| | |
|---|---|
| sb | -0.075 |
| sbs | not used |
| fn | 2048 |

**F1 PROCESSING**

| | |
|---|---|
| gf1 | 0.008 |
| gfs1 | not used |
| proc1 | lp |
| fn1 | 2048 |

**DISPLAY**

| | |
|---|---|
| sp | -408.4 |
| wp | 5341.9 |
| sp1 | -1883.5 |
| wp1 | 30154.5 |
| rfl | 408.4 |
| rfp | 0 |
| rfl1 | 1883.5 |
| rfp1 | 0 |

**PLOT**

| | |
|---|---|
| wc | 142.6 |
| sc | 0 |
| wc2 | 127.1 |
| sc2 | 12.7 |
| vs | 503 |
| th | 1 |
| ai | cdc av |

**ACQUISITION ARRAYS**

| | |
|---|---|
| array | phase |
| arraydim | 512 |
| i | phase |
| 1 | 1 |
| 2 | 2 |

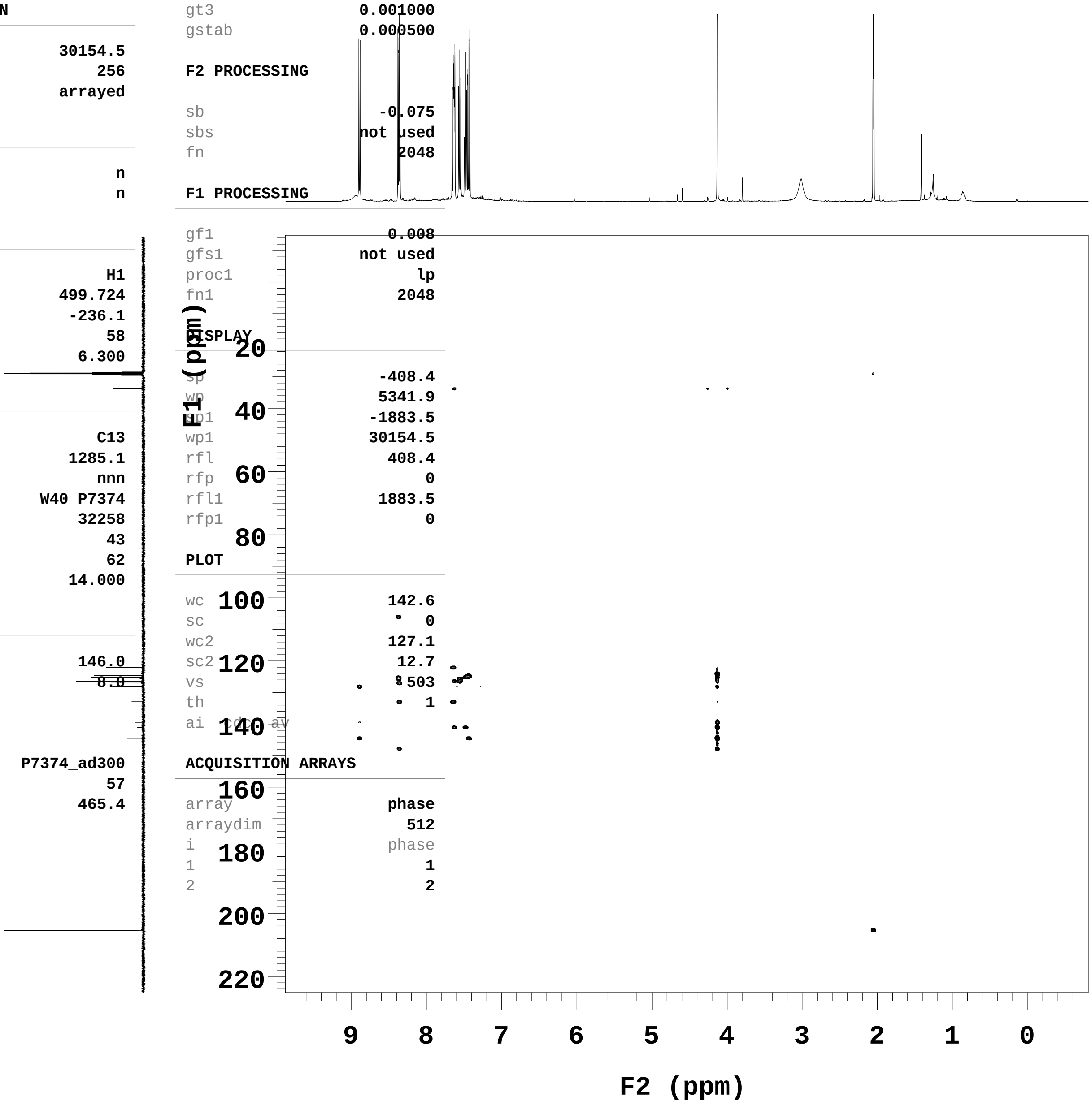


**Plotname: RC-2-C-6-141B_gHMBCAD_20240814_01_plot01**

Data file /home/data/MBaumann/cscb500/RuairiCrawford/2024/20240814_RC-2-C-6-141B_01/RC-2-C-6-141B_gHMBCAD_20240814_01.fid Plot date 2024-08-14
